# Floridoside as a Hinge-Targeted Inhibitor of MAPK13: Atomistic Insights from Molecular Dynamics Simulations

**DOI:** 10.3390/md24060191

**Published:** 2026-05-27

**Authors:** Yang Zhong, Feng Liang, Zhongli Xiong, Zhen Liu

**Affiliations:** 1 School of Chemical Engineering, East China University of Science and Technology, Shanghai 200237, China; y30230029@mail.ecust.edu.cn; 2 Shanghai Zhengxin Biotechnology Co., Ltd., Shanghai 201612, China; liangfeng@zhengxinbiotec.cn

**Keywords:** floridoside, MAPK13, molecular dynamics simulation, binding free energy, binding mechanism

## Abstract

Floridoside (2-(α-D-galactosyl)glycerol) is a compatible solute synthesized in red algae, known for its antioxidant, immunostimulatory, anti-inflammatory, and antimicrobial properties. However, the lack of target validation has limited mechanistic insights into its bioactivity. Mitogen-activated protein kinase 13 (MAPK13), a member of the p38 mitogen-activated protein kinase (p38 MAPK) family with unique structural and functional characteristics, plays an important role in respiratory tissue remodeling, tumor progression, and immune responses, making it an attractive therapeutic target. This study identifies MAPK13 as a high-affinity target of floridoside. In vitro kinase assays validated that floridoside effectively inhibits MAPK13 with a nanomolar inhibitory concentration (IC50 = 13.59 nM), significantly outperforming the classical inhibitor BIRB-796. Unbiased molecular dynamics simulations and steered molecular dynamics simulations reveal that floridoside binds within the MAPK13 hinge region via an ATP-competitive mechanism. Binding free energy analysis combined with computational alanine scanning highlight Asp-113 as a primary interaction hotspot, stabilized by persistent hydrogen bonds with Pro-108 and Met-110. Despite stable complex formation, the flexibility of the glycosidic bond and glycerol tail may limit binding persistence. Comparative simulations with 2-α-glucosylglycerol (2αGG), a stereoisomer of floridoside, demonstrate the sensitivity of MAPK13 binding to subtle structural variations. These findings elucidate the atomistic basis for floridoside’s bioactivity and establish it as a candidate natural scaffold for the design of isoform-selective p38 inhibitors.

## 1. Introduction

**Figure 1:**
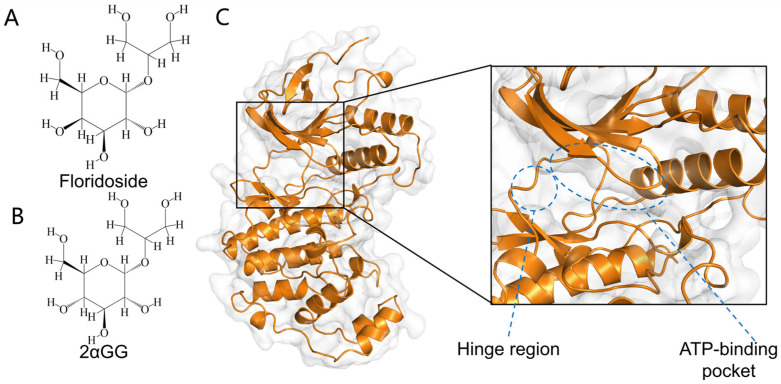
Structure of the (**A**) floridoside, (**B**) 2αGG and (**C**) MAPK13.

Floridoside is a glycoside compound composed of a galactose and a glycerol unit linked via an α-1,2-glycosidic bond (Figure 1A). It is synthesized by red algae, a major group of marine organisms, where it serves as a key compatible solute in cytoplasmic osmoregulation by modulating osmotic pressure [1,2,3]. Experimental studies have demonstrated its multifaceted biological activities, including antioxidative, anti-inflammatory, and immunomodulatory effects [4,5,6]. Importantly, no direct evidence of human toxicity or adverse effects has been reported, supporting its potential for applications in pharmaceutical development, cosmetic formulations, and functional food engineering.

Among the diverse biological activities of floridoside, its anti-inflammatory mechanisms and translational potential have attracted increasing interest. In vitro studies in LPS-activated BV-2 microglial cells demonstrate that this compound exerts anti-inflammatory effects through suppression of mitogen-activated protein kinase (MAPK) signaling pathway, leading to reduced release of pro-inflammatory mediators [4]. Floridoside treatment significantly inhibits the production of nitric oxide and reactive oxygen species, accompanied by downregulation of inducible nitric oxide synthase and cyclooxygenase-2 expression [4,7]. However, while some studies have suggested potential interactions between floridoside and proteins such as TNF-α and SARS-CoV-2 nsp15, its broader molecular mechanisms and specific targets within the human kinome remain largely unexplored [8,9]. In particular, direct evidence of its interaction with specific signaling kinases has been lacking, presenting a critical knowledge gap in understanding how floridoside regulates complex intracellular signaling pathways.

The MAPK signaling pathway serves as a central signaling network that coordinates cellular proliferation, differentiation, apoptosis, and stress responses [10]. Among its branches, the p38 mitogen-activated protein kinase (p38 MAPK) has been extensively studied due to its critical involvement in inflammatory regulation, oxidative stress responses, and DNA damage repair processes [11,12].

Upon activation by environmental stressors or inflammatory stimuli, upstream kinases of the p38 MAPK signaling cascade initiate signaling transduction in response to oxidative stress, osmotic fluctuations, or cytokine signals [13]. This cascade sequentially activates mitogen-activated protein kinase kinase 3 and 6, which phosphorylates p38 MAPK [14]. The phosphorylated p38 MAPK then regulates the activation of multiple downstream effectors, including transcription factors, apoptotic regulators, protein kinases, cell cycle controllers, and autophagy-related proteins, mediating diverse cellular responses [15,16]. As a central regulatory node in cellular stress and inflammatory responses, p38 MAPK is aberrantly activated in various disease states, making it a critical therapeutic target for inflammatory disorders and cancer [17,18].

The p38 MAPK family consists of four isoforms: p38α (MAPK14), p38β (MAPK11), p38γ (MAPK12), and p38δ (MAPK13). Among these, MAPK13 exhibits unique functional properties in the pathogenesis of various diseases, particularly in inflammation, cancer, metabolic disorders, and cardiovascular diseases [19,20,21,22,23]. The structure of MAPK13 contains an N-terminal domain primarily formed by β-sheets that coordinates adenosine triphosphate (ATP) binding and catalytic activity, coupled with a C-terminal domain characterized by α-helical structures that mediate substrate recognition and regulatory interactions [24,25,26]. Comparative structural studies reveal that MAPK13 has a more constrained ATP-binding pocket compared to the well-characterized MAPK14 isoform, resulting from differences in spatial orientation between its N- and C-terminal domains [27]. Furthermore, MAPK13 contains a unique allosteric binding pocket within its C-terminal lobe, where a hydrophobic core stabilized by a network of complementary hydrogen bonds provides the structural foundation for designing isoform-selective inhibitors [21,26,28].

Pharmacological strategies targeting p38 MAPK family involve two primary classes of small-molecule inhibitors: ATP-competitive inhibitors and allosteric modulators. The former directly competes with ATP for binding at the catalytic site, while the latter functions by binding to distal regulatory regions, inducing conformational changes that inhibit enzymatic activity [29]. Current research on MAPK13 inhibitors has primarily focused on synthetic compounds, such as NuP-3, NuP-4 and doramapimod (BIRB-796) [30,31,32,33], featuring a halogenated benzimidazole scaffold to engage the hinge region and a hydrophobic tail group occupies hydrophobic pocket.

Floridoside, however, displays no structural resemblance to existing MAPK13 inhibitors, highlighting the need to elucidate its binding mode and mechanistic engagement with the target.

Interestingly, another natural small-molecule glycoside, glucosylglycerol, has also been reported to show potential therapeutic effects in the areas of inflammation, oxidative stress, and tumor suppression [34,35,36]. The α-anomeric form of glucosylglycerol (2αGG) exhibits significant structural similarity to floridoside (Figure 1B), yet systematic target validation studies for this specific stereoisomer are currently lacking. This provides an opportunity to explore how minor stereochemical differences affect binding behavior and interaction stability.

To address these knowledge gaps, this study combines molecular docking, molecular dynamics simulations and in vitro validation to clarify the binding mode of floridoside within the MAPK13 hinge region. The comparative analysis with 2αGG was conducted to evaluate the structural specificity and dynamics of interaction. The primary objective of this study is to validate the direct inhibitory effects of floridoside on the specified targets and to elucidate their underlying binding mechanisms, thereby providing novel insights for subsequent drug screening and development.

## 2. Results

### 2.1. Modelling and Docking Results

The structural integrity of the protein model, following the repair of missing residues, was assessed using the PROCHECK module via the UCLA-DOE SAVES v6.1 online server. The results indicated that 91.7% of the residues in the repaired model are located within the most favored regions, with 0% in the disallowed regions (Figure S8). These metrics confirm that the model satisfies the criteria for a high-quality protein structure.

**Figure 2:**
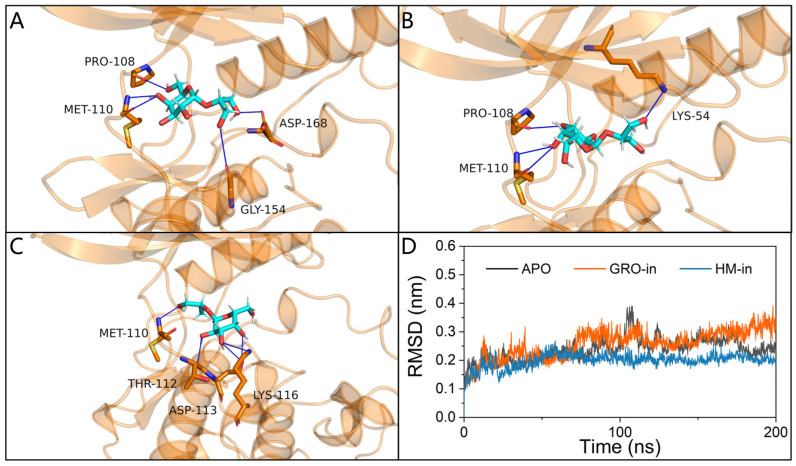
Initial structures of the (**A**) HM-in, (**B**) HM-out and (**C**) GRO-in orientation, and (**D**) RMSDs of the backbone of APO, HM-in and GRO-in system.

Due to the lack of crystallographic data for floridoside–MAPK13 complexes and the considerable structural differences between floridoside and established MAPK13 inhibitors, three top-scoring docking poses were selected for molecular dynamics simulations (Figure 2A–C). These poses exhibit three distinct binding configurations: the hydroxymethyl group on galactose ring pointing inward (HM-in), the hydroxymethyl group pointing outward (HM-out), and the glycerol tail oriented inward (GRO-in). All identified conformations were located within the MAPK13 hinge region. The binding affinities for the three selected poses were −5.1 kcal·mol^−1^ (for GRO-in), −5.0 kcal·mol^−1^ (for HM-in) and −5.0 kcal·mol^−1^ (for HM-out).

PLIP analysis revealed distinct hydrogen-bonding patterns among the floridoside binding modes: (1) the HM-in conformation formed hydrogen bonds with Pro-108, Met-110, Gly-154, and Asp-168; (2) the HM-out conformation formed hydrogen bonds with Pro-108, Met-110, and Lys-54; (3) the GRO-in conformation formed hydrogen bonds with Met-110, Thr-112, Asp-113, and Lys-116. These three binding conformations were subsequently subjected to molecular dynamics simulations to evaluate their dynamic stability and potential variations in binding modes.

### 2.2. Root Mean Square Deviation

To minimize potential artifacts from static docking, 200 ns MD simulations were conducted to characterize system dynamics. The HM-out conformation exhibited ligand dissociation within the first 50 ns and was therefore excluded from further analysis (Figure S1). Subsequent Root Mean Square Deviation (RMSD) calculations for the HM-in and GRO-in conformations revealed structural stability throughout the simulation trajectory (Figure 2D). Both conformations achieved equilibrium after approximately 70 ns of simulation time. During the equilibrium phase, RMSD fluctuations remained below 0.1 nm, suggesting enhanced structural rigidity compared to apoprotein (APO) form. The HM-in conformation demonstrated superior stability relative to GRO-in, as indicated by smaller RMSD fluctuations and earlier convergence. Given satisfactory convergence across systems, trajectories were retained for subsequent analysis.

### 2.3. Root Mean Square Fluctuation

**Figure 3:**
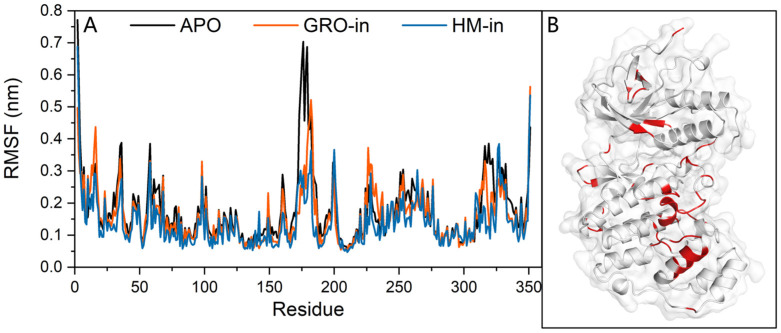
(**A**) RMSF profiles of all systems during the 200 ns MD simulation. (**B**) Visualization of residues exhibiting RMSF variations exceeding 30% compared to the APO system.

Root Mean Square Fluctuation (RMSF) analysis was employed to evaluate residue-specific flexibility and assess ligand-induced alterations in protein dynamics. Compared with the APO form, the HM-in binding mode showed reduced RMSF values in substrate-binding and ATP-binding site, particularly for residues proximal to the catalytic loop and C-terminal α-helix region (Figure 3). These observations suggest enhanced rigidity in these regions. A detailed analysis of ATP-binding site residues revealed a marked reduction in flexibility of Pro-108, Met-110, and Asp-113. These residues were subsequently identified as critical for protein–ligand interactions, providing key hydrogen bonds that stabilize ligand binding.

### 2.4. Binding Free Energy Calculations

The binding free energies of the protein–ligand complexes were calculated using the MM-PBSA method (Table 1). Snapshots were extracted from the last 20 ns of the equilibrium phase for each simulated system. Both conformations exhibited negative enthalpy changes, confirming thermodynamically favorable binding interactions. However, significant differences in absolute binding free energy values were observed. Energy decomposition analysis into Coulombic interactions, van der Waals forces, polar solvation, and nonpolar solvation components revealed that these differences mainly originated from variations in Coulombic terms and PB energy contributions. Polar solvation energy made a significantly unfavorable contribution to the total binding free energy. After entropy correction, the HM-in conformation retained a negative ∆G value (−8.08 ± 1.46 kcal·mol^−1^), whereas the favorable enthalpy change of the GRO-in conformation was offset by entropy penalties, resulting in an overall positive binding free energy (3.19 ± 1.21 kcal·mol^−1^).

Covariance between energy terms was calculated to quantify their interdependencies (Table S7). The total variance of the binding free energy was primarily due to Coulomb energy (61.23 kcal^2^·mol^−2^) and polar solvation energy (31.29 kcal^2^·mol^−2^). However, a pronounced negative covariance (−14.32 kcal^2^·mol^−2^) was observed between ∆E_cou_ and ∆E_PB_.

### 2.5. Binding Mode and Key Residue

**Figure 4:**
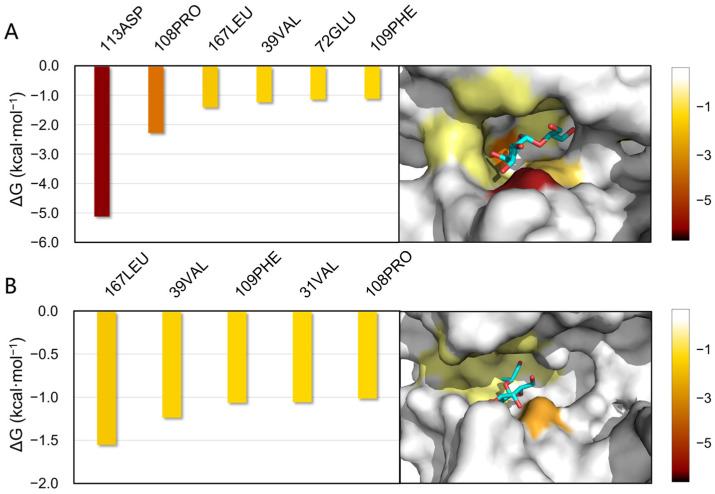
Residue energy decomposition of binding free energy in (**A**) HM-in and (**B**) GRO-in mode. Three-dimensional schematic structures of binding region were extracted from the lowest-energy conformation and colored according to the corresponding residue energy intensities. Hydrogen atoms were hidden to reduce visual clutter and emphasize key intermolecular interactions.

To identify the atomic-level determinants of binding affinity, per-residue energy decomposition analysis of the binding free energy calculations was performed (Figure 4, Tables S1 and S2). In the HM-in binding mode, Asp-113 exhibited the strongest binding contribution (−5.15 kcal·mol^−1^), followed by Pro-108 (−2.31 kcal·mol^−1^) and Leu-167 (−1.45 kcal·mol^−1^). For the GRO-in mode, the primary contributors were Leu-167 (−1.56 kcal·mol^−1^), Val-39 (−1.25 kcal·mol^−1^), and Phe-109 (−1.07 kcal·mol^−1^). In most cases, Coulombic interactions were the dominant contributors to the total binding energy.

**Figure 5:**
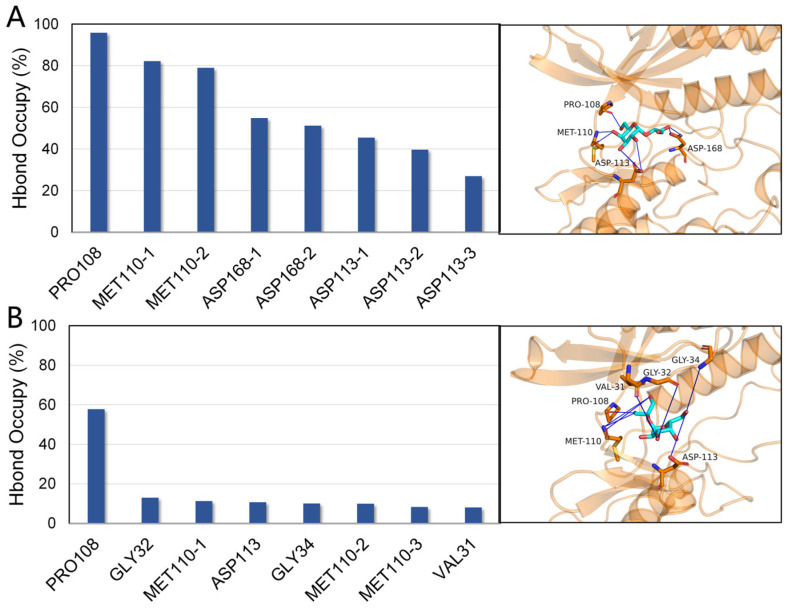
Hydrogen bond occupancy percentages in (**A**) HM-in and (**B**) GRO-in mode. Three-dimensional schematic structures of binding region were extracted from the lowest-energy conformation, with blue solid lines representing hydrogen bonds exhibiting higher temporal persistence. The solid connectors solely indicate interatomic relationships and do not represent actual bonding patterns. Hydrogen atoms were hidden to reduce visual clutter and emphasize key intermolecular interactions.

Hydrogen bond occupancy analysis was carried out based on the last 100 ns of the equilibrated trajectories. The top 8 stable hydrogen bonds in both HM-in and GRO-in binding modes were highlighted (Figure 5A). Typically, hydrogen bonds with occupancies exceeding 50% are considered stable, whereas those exceeding 80% of occupancies are regarded as highly stable.

The HM-in conformation maintained multiple persistent hydrogen bond interactions (Figure 5A, Table S4). The O6 and O8 hydroxyl oxygen on the galactose ring of floridoside formed highly stable hydrogen bonds with Pro-108 and Met-110, with occupancies exceeding 80%. Additional stable interactions meeting the threshold criterion involved Asp-168 and Asp-113. In contrast, the GRO-in conformation maintained only a single moderately stable hydrogen bond as its principal interaction (Figure 5B, Table S5).

Noting the putative functional role of Asp-113 in molecular recognition, computational alanine scanning (CAS) was performed by mutating Asp-113 to alanine (designated D2A) [37], followed by a binding free energy recalculation (Table 1 and Table S9). The mutation caused a 64.4% reduction in binding affinity, as reflected by the decrease in the magnitude of the binding free energy. The Coulombic interaction energy exhibited a marked reduction, attributable to replacement of the carboxyl group in aspartate with the nonpolar methyl side chain of alanine, thereby abolishing the hydrogen bonds between Asp-113 and floridoside. Van der Waals interactions decreased slightly, due to reduced steric hindrance from the smaller side chain of alanine compared to that of aspartate.

### 2.6. Dynamic Cross-Correlation Matrix

**Figure 6:**
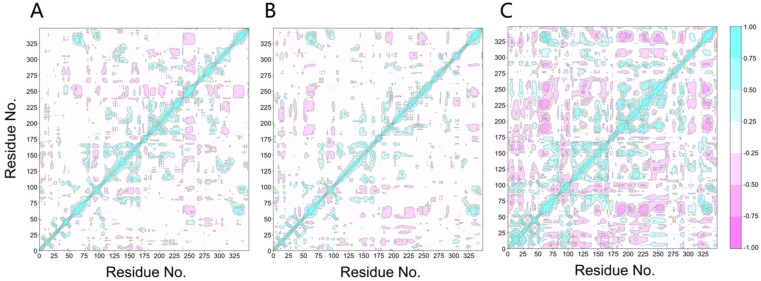
Cross-correlation matrixes for Cα atoms in (**A**) APO, (**B**) HM-in and (**C**) GRO-in system. Red and blue regions indicate positively and negatively correlated motions of Cα atoms respectively.

A dynamic cross-correlation matrix was constructed based on Cα atom fluctuations at the residue level and helped to understand the effects of ligand on protein conformational dynamics (Figure 6). Red and blue regions indicated positively and negatively correlated motions of Cα atoms, reflecting collective residue-level dynamics. Compared with the APO system, in HM-in system, the binding enhances the cooperativity between the N-lobe and the N-terminus of the C-lobe of MAPK13, while simultaneously uncoupling the conformational relationship between the C-terminal region of the C-lobe and the rest part of the C-lobe. While the GRO-in binding mode was thermodynamically less favorable, the magnitude of perturbations within its cross-correlation matrices was significantly greater.

### 2.7. Free Energy Landscapes

**Figure 7:**
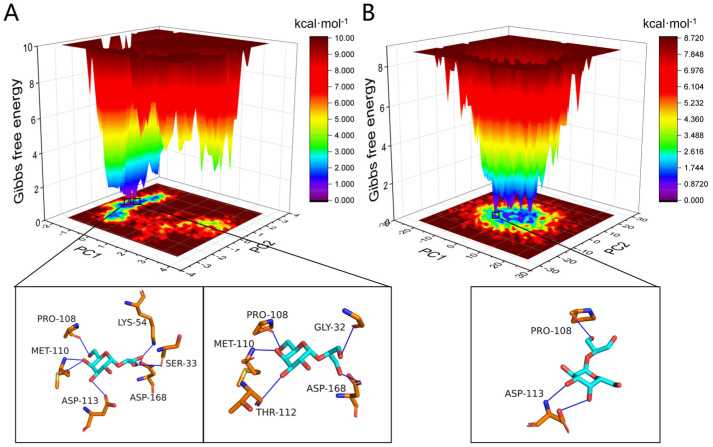
Three-dimensional free energy landscapes from MD trajectories for (**A**) HM-in and (**B**) GRO-in mode, with purple-shaded regions indicating the lowest-energy conformations. Hydrogen atoms were hidden to reduce visual clutter and emphasize key intermolecular interactions.

Free energy landscapes were constructed based on principal component analysis. The covariance matrix was generated from atomic fluctuations within 10 Å of floridoside, and the resulting trajectory was projected onto the first two principal components (PC1 and PC2) (Figure 7).

The free energy landscape of the HM-in binding mode showed a global minimum (purple basin) and a secondary minimum, corresponding to two thermodynamically stable binding substates. The narrow separation between these minima suggested high structural rigidity.

Structural characterization of the energy minima revealed that the two HM-in substates were differentiated by the rotation angles of the glycerol tail, resulting in distinct hydrogen-bonding patterns. The concentrated distribution of energy minima indicates a consistent overall binding mode, despite local conformational variations.

In contrast, the GRO-in binding mode exhibited a global free energy minimum accompanied by multiple dispersed sub-minima with comparable energy levels. Notably, several trajectories showed decrease in the number of hydrogen bonds and a collapse of local binding geometry, corroborating the reduced binding energy.

### 2.8. SMD

**Figure 8:**
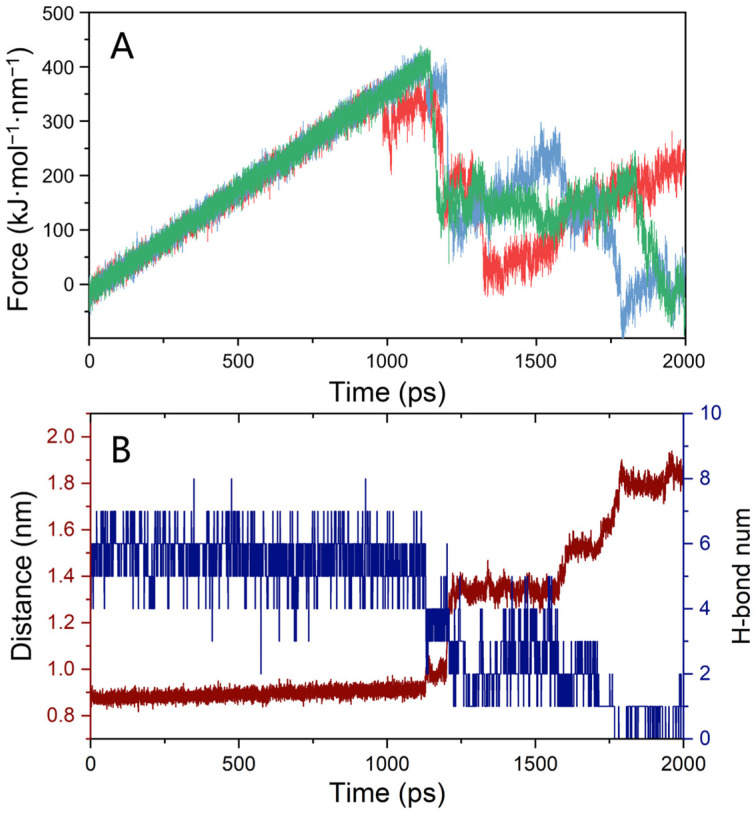
(**A**) Force applied to floridoside within the three SMD simulations. Green, blue, and red lines represent three independent simulation replicates. (**B**) Relation between distance and H-bond number during the simulation. Blue and red lines represent the number of H-bonds and the distance between the ligand and the binding pocket, respectively.

SMD simulations were used to investigate ligand dissociation processes by quantifying the external force required to displace a ligand from the protein’s hinge region, thereby providing a basis for assessing binding affinity from a different perspective. The HM-in conformation, identified as the dominant binding mode, was selected for SMD analysis to examine the dissociation mechanism and energetic characteristics of floridoside from the MAPK13 hinge region (Figure 8). A virtual damped harmonic spring model was employed, with a constant pulling velocity of 5 × 10^−4^ nm·ps^−1^ and a spring constant of 800 kJ·mol^−1^·nm^−2^.

Since the natural dissociation pathway was unknown, ten independent SMD simulations were performed with pulling forces applied along the X, Y, and Z directions to cover the main dissociation paths as much as possible. At the beginning of each simulation, the force increased gradually as the ligand resisted displacement. Once the applied force surpassed a critical threshold, the ligand began to dissociate from the binding pocket. Among these trajectories, the three with the lowest rupture forces were selected to represent the most probable natural dissociation pathways, yielding an average maximum force of 415.81 ± 22.46 kJ·mol^−1^·nm^−1^, indicative of a considerable mechanical barrier to ligand dissociation. The average peak force across all ten simulations was 458.88 ± 43.68 kJ·mol^−1^·nm^−1^, which reflected the overall mechanical response of the system (Figure S2).

### 2.9. Inhibitory Efficacy of Floridoside Against MAPK13

**Figure 9:**
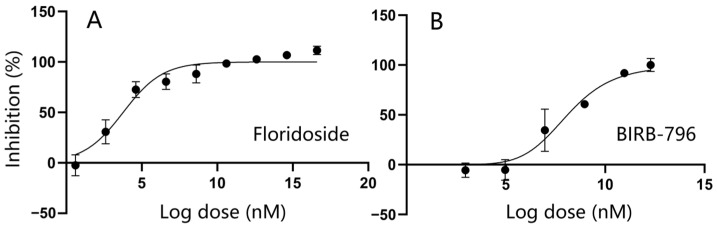
Dose–response curve of (**A**) floridoside and (**B**) BIRB-796 inhibiting MAPK13 activity. Inhibition of MAPK13 by floridoside was measured at various concentrations. The x-axis represents the logarithmic concentration of inhibitors, and the y-axis shows the percentage of inhibition. Data points are expressed as mean ± SD (*n* = 3). The IC50 value was determined by fitting the data to a non-linear regression model.

The inhibitory activity of floridoside against MAPK13 was quantitatively evaluated using the ADP-Glo luminescence assay (Table S10). Floridoside demonstrated a dose-dependent inhibition of MAPK13 kinase activity. As shown in the dose–response analysis, the relative activity of the kinase decreased monotonically as the concentration of floridoside increased (Figure 9). Non-linear regression fitting yielded a half-maximal inhibitory concentration IC50 of 13.59 nM, with a high coefficient of determination (R^2^ = 0.9290), indicating a robust and high-affinity interaction between floridoside and the target protein. For BIRB-796, non-linear regression analysis yielded an IC50 of 274.58 nM and an R^2^ of 0.9417.

### 2.10. Comparative Perspectives on Ligand Binding Behavior

MD simulations of 2αGG employed the same protocols and analytical methodologies as those used for floridoside. Despite the structural similarity between the two compounds, the opposite orientation of the methyl group at the C3-position of the galactose ring resulted in distinct binding modalities (Figure S3).

Docking results revealed that the O5 and O7 atoms of 2αGG were significantly displaced from Asp-113 relative to their positions in the floridoside complex. Molecular dynamics trajectories further demonstrated reorientation of the glycerol tail, with the O4 atom shifting inward to form a hydrogen bond with Pro-108 (occupancy: 93.353%, Table S6). The original O6-Pro-108 hydrogen bond was disrupted, and a new multi-dentate hydrogen-bonding interaction emerged between O6 and Met-110 (Figure S4). Although the galactose ring of 2αGG exhibited outward displacement to establish contact with Asp-113, the hydrogen-bond occupancy between them remained below 10%. In contrast, floridoside maintained a total occupancy of 130% with this residue (Table S6).

Spontaneous conformational adjustments in the 2αGG-MAPK13 complex shifted its binding mode toward the GRO-in conformation. Binding free energy data and residue energy decomposition revealed weakened Coulombic interactions in the 2αGG-MAPK13 complex (−17.93 ± 1.76 kcal·mol^−1^), primarily due to the loss of key interactions with Asp-113, thereby reducing the overall binding enthalpy (−11.62 ± 1.11 kcal·mol^−1^) (Table 2 and Table S3, Figure S5). Covariance calculations revealed a significant negative correlation between coulombic energy and polar solvation free energy, showing a compensatory effect between these two terms (Table S8). The free energy landscape showed a primary deep basin, demonstrating convergence within the conformational ensemble (Figure S6).

Although 2αGG maintained stable positioning within the binding pocket throughout the 200 ns simulation trajectory, its higher binding free energy and reduced hydrogen-bond count collectively indicated compromised binding affinity.

## 3. Discussion

Molecular dynamics simulations provide deep insight into the stability of different floridoside binding poses, with multiple analyses consistently identifying the HM-in conformation as the thermodynamically and structurally preferred binding mode. Binding free energy calculations show that the HM-in conformation possesses significantly more favorable thermodynamic interactions, with a strong binding affinity driven by a large negative enthalpy change, which facilitates a superior hydrogen bond network dominated by a multi-dentate engagement with Asp-113 and stabilized by Pro-108 and Met-110. The quantity and spatial distribution of these hydrogen bonds show strong concordance with molecular docking predictions.

In contrast, the GRO-in conformation orients its highly polar ring toward the solvent interface, incurring substantial polar solvation energy penalties that account for its weaker binding. The free energy landscapes visually corroborate this distinction: the HM-in system is characterized by a focused, deep energy basin indicative of a constrained and stable conformational ensemble, whereas the GRO-in landscape is flat and dispersed, suggesting rapid interconversion among loosely bound sub-states rather than a single dominant pose.

While the thermodynamic picture is clear, a detailed analysis reveals important complexities. The ligand’s high polarity incurs significant desolvation costs, resulting in unfavorable polar solvation energy. Since the standard deviation of enthalpy exceeded the 15 kJ·mol^−1^ convergence threshold, calculated entropy penalties (−TΔS) are likely overestimated [38]. Nevertheless, the HM-in system’s large negative enthalpy ensures highly favorable binding free energy, reinforcing our conclusion. Furthermore, a strong anticorrelation between Coulombic and polar solvation energies reflects a dynamic compensation effect, where direct electrostatic interactions and solvent-mediated shielding are in constant balance. While this reduces cumulative statistical uncertainty, it amplifies instantaneous free energy fluctuations.

From a kinetic perspective, although SMD simulations cannot provide the ligand’s residence time, it revealed a pronounced resistance to unbinding for the HM-in pose, corroborating its robust retention within the hinge pocket and consistent with the thermodynamic findings. Dynamic cross-correlation analysis further suggests that the HM-in mode can perturb the protein through a more localized and thermodynamically efficient mechanism, whereas the GRO-in mode induces more pervasive conformational reorganization at a greater energetic cost.

A deeper analysis of floridoside’s binding mode, when contextualized with the extensive literature on MAPK13 inhibitors, underscores its distinct mechanism. Pharmacological strategies targeting the p38 MAPK family often involve Type II inhibitors, which stabilize an inactive DFG-out conformation and engage a unique allosteric site adjacent to the ATP pocket [32]. A classic example is an inhibitor like NuP-3, whose large scaffold physically spans all the regions to lock the kinase in this inactive state, including the hinge, the ATP-binding site, and the allosteric pocket [31]. In our unbiased simulations, floridoside remained stably anchored within the hinge region, and no significant contact with the distal allosteric pocket or the characteristic DFG motif flip was observed. This suggests that floridoside functions as a putative ATP-competitive (Type I) inhibitor, a mechanism fundamentally different from these well-documented allosteric modulators. This distinction also extends to its chemical interactions. Unlike potent synthetic inhibitors that use specific hydrogen bonds to anchor in the hinge region while leveraging large hydrophobic tails to maximize favorable van der Waals contacts in deeper pockets [33], floridoside’s affinity is driven almost exclusively by a dense network of hydrogen bonds mediated by its polyhydroxy structure, which also incurs a significant desolvation penalty. Furthermore, the structural nuances between p38 isoforms are critical for inhibitor design. It has been established that MAPK13 possesses a more constrained ATP-binding pocket compared to the canonical MAPK14 isoform [27]. The compact and highly polar nature of floridoside may be particularly well-suited to this more restricted geometry, which provides a plausible structural rationale for why a compact, polar ligand such as floridoside may show relative isoform preference despite lacking the hydrophobic appendages common to potent NuP-4-like inhibitors.

In vitro assays identified floridoside as a modulator of MAPK13, demonstrating inhibitory activity in the low nanomolar range and surpasses that of the classical inhibitor BIRB-796 (IC50 ≈ 274.58 nM). The strong inhibitory potency observed in vitro is consistent with the highly negative binding free energies predicted by our simulations, suggesting that the identified binding mode is energetically favorable. The SMD rupture force of ~415 kJ·mol^−1^·nm^−1^ also reflects a stable binding interface under mechanical stress. These findings offer a plausible molecular-level explanation for the previously reported inhibitory effects of floridoside on the MAPK pathway [4], and we propose that its documented anti-inflammatory activity is driven, at least in part, by this direct inhibition of MAPK13.

Furthermore, the identification of MAPK13 as a high-affinity target complements earlier in silico reports exploring floridoside’s broader interactome. In silico studies have suggested that floridoside may occupy the active pocket of the TNF-α trimer, establishing a robust interaction network with residues Tyr119, Pro117, and Glu116 through a combination of hydrogen bonds and alkyl hydrophobic interactions [9]. This shared involvement in inflammatory signaling pathways suggests a possible synergistic effect. Beyond inflammation, floridoside is also predicted to target the catalytic core of the SARS-CoV-2 nsp15 endoribonuclease, forming critical hydrogen bonds with Lys290, Ser294, and His235, as well as van der Waals interactions with His250 [8]. The recurrent pattern, where floridoside utilizes its polyhydroxyl scaffold to form dense, site-specific hydrogen bond networks, underscores the versatility of its hydrogen-bonding network in achieving precise molecular recognition across diverse protein architectures. The potential of floridoside for synergistic multi-node regulation of signaling pathways further emphasizes its value as a lead scaffold for developing multi-target anti-inflammatory agents.

Analysis of key residue interactions provides clear insights for rational drug design. With Asp-113 confirmed as the primary hotspot, future optimization should focus on strengthening hydrogen bonds to it and Pro-108. Given the dominance of electrostatic interactions and the high desolvation penalty, another viable strategy is the judicious introduction of hydrophobic groups at the terminal glycerol positions (e.g., C1 or C3 methylene groups) to enhance van der Waals contacts and potential π-π stacking. The goal of such modifications would be to push ΔG lower, approaching the binding strengths of state-of-the-art δ-selective inhibitors and boosting the efficacy of floridoside analogues in suppressing MAPK13-driven pathological processes. As this kinase regulates overproduction of matrix metalloproteinases and pro-inflammatory cytokines in dermatological contexts [16], the detailed atomic interactions illustrated here provide a foundation for targeting cutaneous inflammatory cascades. Optimized derivatives hold promise for reducing UV-induced MMP-1 activation and IL-6 secretion [16], which are key mediators of collagen degradation and skin barrier impairment.

Finally, a comparative analysis with the structural analog 2αGG highlights the exquisite sensitivity of the binding interaction. Despite sharing similar interaction patterns with MAPK13, primarily with Pro-108 and Met-110, a subtle difference in the orientation of a methyl group at the C3-position of the galactose ring results in a complete lack of thermodynamic affinity for 2αGG. This underscores how minor conformational variations can profoundly impact binding and emphasizes the critical importance of precise structural matching in molecular recognition.

## 4. Materials and Methods

### 4.1. Structure Modeling

The crystal structure of MAPK13 was obtained from the RCSB Protein Data Bank (PDB ID: 4YNO), with missing residues subsequently reconstructed using Modeller version 10.7 (Figure 1C) [39]. The PROCHECK module via the UCLA-DOE SAVES v6.1 online server was used to assess the structural integrity of the protein model. The molecular geometries of floridoside and 2αGG were fully optimized at the density functional theory level using Gaussian 09 [40], employing the B3LYP functional [41] with Grimme’s dispersion correction [42] and the def2-SVP basis set.

### 4.2. Molecular Docking

Semi-flexible docking was conducted using AutoDock Vina version 1.2.5 [43], with the binding site encompassing the hinge region and the hydrophobic pocket of MAPK13. The docking grid box was centered at coordinates (33.338, −37.118, 3.167) with dimensions of 3 × 3 × 3 nm^3^. The iterated local search algorithm was employed, incorporating gradient-based BFGS optimization with a hybrid scoring function that integrates empirical parameters and knowledge-based potentials for binding affinity prediction [44]. Based on the top-ranked docking conformations, three distinct floridoside binding modes were selected for subsequent MD simulations: one with the hydroxymethyl group (HM) of the galactose ring oriented outward (HM-out), another with the HM oriented inward (HM-in), and a third with the glycerol tail (Gro) adopting an inward orientation (GRO-in). For 2αGG, HM-in mode was selected due to its high binding affinity.

### 4.3. Molecular Dynamics Simulation

Based on the initial structure obtained from molecular docking, molecular dynamics simulations were performed using the GROMACS 2024 software package [45]. The protein was processed and protonated using the pdb2gmx module within GROMACS, employing the CHARMM36 all-atom force field [46]. For the ligand, parameters were generated via the CHARMM General Force Field scheme, with partial atomic charges assigned automatically based on chemical analogy [47]. The solvated system was built in a cubic simulation box (10 × 10 × 10 nm^3^) under periodic boundary conditions, using TIP3P water molecules. System charge neutrality was ensured by adding counterions, with NaCl concentration adjusted to 0.15 M to mimic physiological conditions. Energy minimization was carried out using the steepest descent algorithm followed by the conjugate gradient method to remove unfavorable interactions or steric clashes.

Positional restraints were applied to protein backbone atoms and ligand non-hydrogen atoms during two sequential equilibration phases. The first phase including a 1 ns NVT ensemble with temperature maintained at 300 K using the V-rescale thermostat [48]. This was followed by a 1 ns NPT ensemble, where the temperature was kept at 300 K using the V-rescale thermostat, and the pressure was maintained at 1 bar using the Berendsen barostat [49]. Unrestrained MD production simulations were then performed for 200 ns under NPT condition (300 K, 1 bar), using the V-rescale thermostat and Parrinello-Rahman barostat [50].

Three independent simulations conducted for each type of initial conformation, with trajectory data saved every 10 ps for subsequent analysis. All simulations employed a 2-fs integration timestep with constraints on bonds involving hydrogen atoms implemented via the Linear Constraint Solver algorithm. Short-range van der Waals interactions were calculated using a 1.0 nm cutoff. Long-range electrostatics were computed using the Particle Mesh Ewald (PME) method [51] with a Fourier grid spacing of 0.16 nm and fourth-order polynomial interpolation. A Coulomb cutoff of 1.2 nm was applied to ensure adequate resolution for the PME algorithm.

### 4.4. Binding Free Energy

The free energy of binding was calculated using the Molecular Mechanics Poisson–Boltzmann Surface Area (MM-PBSA) method [38]. The calculated binding free energy (∆G_bind_) is decomposed into three primary components: the gas-phase molecular mechanical energy (∆E_MM_), the solvation free energy change (∆G_solv_) and the entropy change (−T∆S), summarized by the equation:

∆G_bind_ = ∆E_MM_ + ∆G_solv_ − T∆S (1)

The molecular mechanical energy was computed with Debye-Hückel screening to account for electrostatic shielding effects in ionic solutions.

The gas-phase energy includes contributions from electrostatic interactions (∆E_COU_), and van der Waals forces (∆E_vdw_), while covalent bond energy terms are neglected due to their negligible contribution to binding energetics. Thus, the molecular mechanical energy is given by:

∆E_MM_ = ∆E_COU_ + ∆E_vdw_ (2)

The solvation free energy term is decomposed into polar (∆G_PB_) and nonpolar (∆G_SA_) components, which can be expressed as:

∆G_solv_ = ∆G_PB_ + ∆G_SA_ (3)

The polar solvation energy is computed by solving the Poisson–Boltzmann (PB) equation, which models the electrostatic potential in a dielectric continuum representing the solvent. Nonpolar contributions are calculated using solvent-accessible surface area (SASA) models that estimate cavity formation and dispersion effects based on molecular surface exposure.

Entropy was calculated using the interaction entropy method based on the final 20 ns of the trajectories. This method assumes that interaction energy distributions follow Gaussian statistics, where large standard deviations in energy values could lead to significant estimation errors [52]. Previous studies have shown that MM-PBSA can provide acceptable accuracy without considering entropy terms [53,54,55,56,57]. Therefore, although the entropy values obtained in this study are reported for reference, the binding free energy analysis focuses on the enthalpic component (ΔH), i.e., ∆E_MM_ and ∆G_solv_.

### 4.5. Trajectory Analysis

Processing and analysis of molecular dynamics trajectories were performed using the integrated toolkit in the GROMACS 2024 package. Protein–ligand interaction patterns were characterized using the Protein–Ligand Interaction Profiler (PLIP) platform [58]. Hydrogen bond formation statistics were quantified using Visual Molecular Dynamics version 1.9.3 [59], based on the R-α(3.5–30) geometric criterion, defined by a donor-hydrogen-acceptor angle ≤ 30° and a hydrogen-acceptor distance ≤ 0.35 nm.

### 4.6. Steered Molecular Dynamics Simulations

To preliminarily estimate the residence time of floridoside within the protein binding pocket, steered molecular dynamics (SMD) simulations were performed by applying an external force to induce ligand dissociation [60]. The final frame of a stable trajectory was extracted from a 200 ns unbiased molecular dynamics simulation and used as the starting structure. SMD simulations were carried out using the GROMACS 2024 software package for 2 ns, with a pulling velocity of 0.0005 nm·ps^−1^. A harmonic spring constant of 800 kJ·mol^−1^·nm^−2^ was applied, and the pulling was conducted along the vector defined by the distance reaction coordinate. To account for variability in possible dissociation pathways and reduce statistical uncertainty, 10 independent SMD replicates were performed.

### 4.7. In Vitro Validation of MAPK13 Inhibition

The inhibitory potential of floridoside against recombinant human MAPK13 (Wild-Type) was evaluated using the ADP-Glo™ Kinase Assay kit (Promega, Madison, WI, USA) [61]. The enzymatic reactions were performed in a 1× reaction buffer consisting of 50 mM HEPES (pH 7.5), 10 mM MgCl_2_, 1 mM DTT, and 0.01% Tween-20. Prior to the primary inhibition studies, a preliminary experiment was conducted to define the linear range of the assay, confirming that at an enzyme concentration of 50 nM, the generation of ADP remained linear with respect to time (30 min) and did not reach signal saturation.

Floridoside was prepared as a 1 mM stock solution and serially diluted to create an 8-point concentration gradient, ranging from 0.0015 μM to 100 μM. The assay was executed in 96-well white opaque plates with a total reaction volume of 25 μL. Each well received 5 μL of floridoside (or solvent for control groups), 15 μL of a mixture containing MAPK13 and the substrate myelin basic protein (MBP), and the reaction was initiated by adding 5 μL of ATP to a final concentration of 50 μM. BIRB-796 served as the control group, with a 6-point concentration gradient ranging from 0.0078 μM to 5 μM. The assay for BIRB-796 was performed under the exact same experimental conditions as those used for floridoside.

Luminescence signals were recorded using a microplate reader after the addition of ADP-Glo™ reagents in accordance with the manufacturer’s protocol. All experimental data were expressed as mean ± standard deviation. The raw luminescence values were normalized to a percentage of relative activity, where the maximum activity control (no inhibitor) was set at 100% and the blank (no enzyme) at 0%. Dose–response curves were modeled using non-linear regression with a four-parameter logistic equation in GraphPad Prism 10.1.2 to calculate IC50 values.

## 5. Conclusions

This study identifies MAPK13 as a molecular target of floridoside through a combination of biochemical assays and molecular dynamics simulations. The results demonstrate that floridoside acts as a candidate ATP-competitive inhibitor with an IC50 of 13.59 nM, which is significantly more effective than classical synthetic inhibitors. The atomic-level analysis reveals that the stability of the floridoside–MAPK13 complex is driven by a hydrogen-bonding network within the hinge region, particularly involving the energy hotspot residue Asp-113 with a contribution of −5.15 kcal·mol^−1^ to ΔG and exhibiting 64.4% binding affinity loss upon mutation. The marked difference in binding affinity between floridoside and its stereoisomer 2αGG underscores the high structural specificity required for MAPK13 inhibition. These findings not only provide a mechanistic explanation for the previously reported anti-inflammatory effects of floridoside but also offer a new candidate natural scaffold for the design of p38 inhibitors. Future studies focusing on cellular target engagement and X-ray crystallography will be essential to further validate these interactions for clinical translation.

## Figures and Tables

**Table 1:** Binding free energies in system HM-in, GRO-in and HM-in with computational alanine scanning by MM-PBSA method (kcal·mol^−1^).

Contribution	HM-in	GRO-in	HM-in (CAS) ^h^
∆E_cou_^a^	−51.35 ± 1.71	−35.49 ± 2.49	−25.10 ± 1.51
∆E_vdw_^b^	−23.57 ± 0.60	−22.68 ± 0.62	−26.33 ± 0.53
∆E_PB_^c^	59.06 ± 1.22	48.72 ± 1.89	48.52 ± 1.24
∆E_SA_^d^	−4.39 ± 0.02	−4.21 ± 0.04	−4.32 ± 0.02
∆H ^e^	−20.26 ± 1.46	−13.64 ± 1.21	−7.22 ± 1.30
−T∆S ^f^	12.18	16.83	14.95
ΔG ^g^	−8.08 ± 1.46	3.19 ± 1.21	7.73 ± 1.30

^a^ Coulombic energy. ^b^ Van der Waals energy. ^c^ Polar solvation free energy. ^d^ Non-polar solvation free energy. ^e^ Total enthalpy change (consisting of molecular mechanics and solvation energy). ^f^ Entropic contribution. ^g^ Binding free energy. ^h^ HM-in system with computational alanine scanning.

**Table 2:** Binding free energies in HM-in and 2αGG-MAPK13 system by MM-PBSA method (kcal·mol^−1^).

Contribution	HM-in	2αGG-MAPK13
∆E_cou_	−51.35 ± 1.71	−17.93 ± 1.76
∆E_vdw_	−23.57 ± 0.60	−25.55 ± 0.53
∆E_PB_	59.06 ± 1.22	36.08 ± 1.20
∆E_SA_	−4.39 ± 0.02	−4.21 ± 0.03
∆H	−20.26 ± 1.46	−11.62 ± 1.11
−T∆S	12.18	13.75
ΔG	−8.08 ± 1.46	2.13 ± 1.11

## Data Availability

The original contribution of this research is included in the article and the Supplementary Materials. Further data underlying this study, including initial structures, ligand parameters, and input files for molecular docking (AutoDock Vina), molecular dynamics simulations (GROMACS 2024), and subsequent analyses (MM-PBSA, RMSD, RMSF, etc.), are available in a Zenodo repository. The data can be accessed at https://doi.org/10.5281/zenodo.15862561. All software used is publicly available as cited in the Section 4.

## Supplementary Materials

The following supporting information can be downloaded at: https://www.mdpi.com/article/10.3390/md24060191/s1, Figure S1: RMSD of floridoside and atoms within 1 nm of the ligand in HM-out system; Figure S2: Force applied to floridoside within 10 SMD simulation; Figure S3: (A) Differences in docking results between 2αGG and floridoside. (B) Initial structure of the binding site and (C) average structure after conformational changes during simulation. (D) RMSD of the backbone in 2αGG-MAPK13 system; Figure S4: Hydrogen bond occupancy percentages in 2αGG-MAPK13 system; Figure S5: Residue energy decomposition of binding free energy in 2αGG-MAPK13 system; Figure S6: 2D free energy landscapes from MD trajectories for 2αGG-MAPK13 system; Figure S7: Cross-correlation matrixes for Cα atoms in 2αGG-MAPK13 system; Figure S8: Ramachandran plot of the MAPK13 structure with repaired residues. 91.7% of the residues in the repaired model are located within the most favored regions, with 0% in the disallowed regions; Table S1: Top 15 energy-contributing residues in the HM-in conformation from residue energy decomposition analysis; Table S2: Top 15 energy-contributing residues in the GRO-in conformation from residue energy decomposition analysis; Table S3: Top 15 energy-contributing residues in the 2αGG-MAPK13 system from residue energy decomposition analysis; Table S4: Top 10 Residues with Highest Hydrogen Bond Occupancy in HM-in Mode; Table S5: Top 10 Residues with Highest Hydrogen Bond Occupancy in GRO-in Mode; Table S6: Top 10 Residues with Highest Hydrogen Bond Occupancy in 2αGG-MAPK13 system; Table S7: Covariance analysis results in HM-in mode; Table S8: Covariance analysis results in 2αGG-MAPK13 system; Table S9: Top 15 energy-contributing residues in the HM-in conformation from alanine scanning and residue energy decomposition analysis; Table S10: Inhibitory effect of Floridoside on MAPK13 activity measured by OD490.
