# Supplementary material for: Floridoside as a Hinge-Targeted Inhibitor of MAPK13: Atomistic Insights from Molecular Dynamics Simulations
Source: Mar Drugs. 2026 May 27;24(6):191. doi: 10.3390/md24060191 (PMC13302522; doi:10.3390/md24060191)
Supplement: Supplementary file 1 [file marinedrugs-24-00191-s001.zip › marinedrugs-4297061-supplementary.pdf]

Supporting Information for

Floridoside as a Hinge-Targeted Inhibitor of MAPK13: Atomistic Insights from Molecular Dynamics Simulations

Yang Zhong[1], Feng Liang[2], Zhongli Xiong [2,*], and Zhen Liu[1,*]

[1] School of Chemical Engineering, East China University of Science and Technology, Shanghai, 200237, China

[2] Shanghai Zhengxin Biotechnology Co., LTD, Shanghai, 201612, China

*Correspondence: xzl@reshy.cn; liuzhen@ecust.edu.cn

1. Root Mean Square Deviation (RMSD)

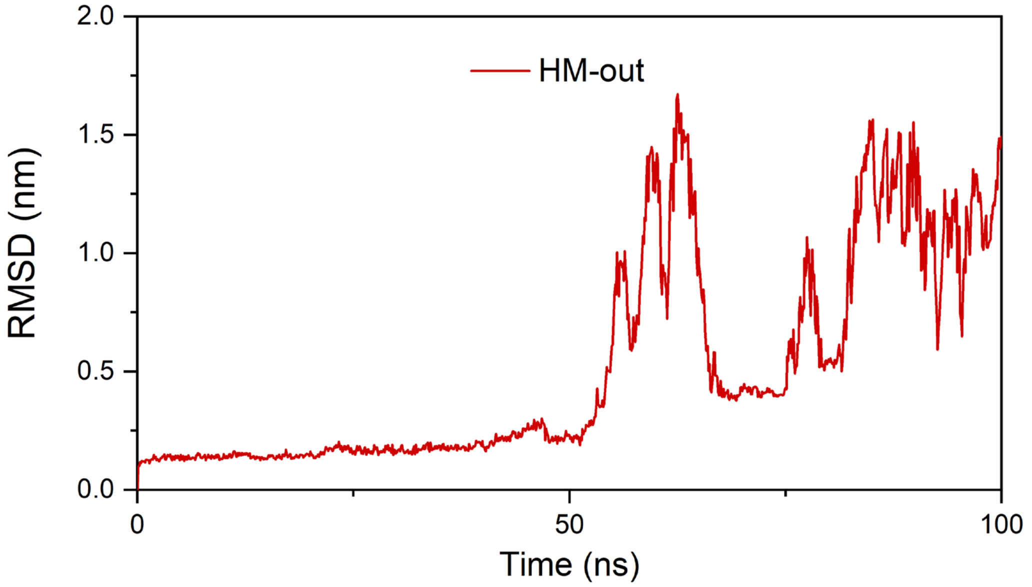

Figure S1. RMSD of floridoside and atoms within 1nm of the ligand in HM-out system.

2. Energy Decomposition

Table S1. Top 15 energy-contributing residues in the HM-in conformation from residue energy decomposition analysis (kcal/mol).

Residue	ΔE_{COU}	ΔE_{vdw}	ΔE_{PB}	ΔE_{SA}	ΔG
113ASP	-12.70	0.93	6.81	-0.20	-5.15
108PRO	-3.16	0.07	0.79	-0.02	-2.31
167LEU	0.30	-1.57	0.08	-0.25	-1.45
39VAL	-0.15	-1.23	0.28	-0.18	-1.27
72GLU	-0.05	-0.03	-1.09	0.00	-1.18
109PHE	-0.93	-1.31	1.15	-0.06	-1.15
110MET	-2.14	-1.30	2.58	-0.10	-0.96
31VAL	-0.03	-1.09	0.55	-0.18	-0.75
107MET	0.13	-0.72	0.01	-0.07	-0.66
32GLY	-0.41	-0.52	0.38	-0.05	-0.60
157ALA	0.03	-0.55	0.04	-0.02	-0.49
154GLY	-0.15	-0.09	-0.10	-0.03	-0.37
89ASP	-0.06	0.00	-0.29	0.00	-0.35
111GLN	0.00	-0.23	-0.09	0.00	-0.33
33SER	-0.16	-0.60	0.53	-0.08	-0.30

Table S2. Top 15 energy-contributing residues in the GRO-in conformation from residue energy decomposition analysis (kcal/mol).

Residue	ΔE_{COU}	ΔE_{vdw}	ΔE_{PB}	ΔE_{SA}	ΔG
167LEU	-0.04	-1.28	0.02	-0.26	-1.56
39VAL	-0.28	-1.05	0.24	-0.16	-1.25
109PHE	-0.72	-1.04	0.73	-0.04	-1.07
31VAL	-0.82	-0.88	0.79	-0.16	-1.07
108PRO	-1.79	-0.14	0.92	-0.02	-1.03
107MET	-0.16	-0.85	0.13	-0.09	-0.98
110MET	-2.21	-1.09	2.69	-0.14	-0.75
52ALA	-0.15	-0.64	0.18	-0.06	-0.67
32GLY	-0.35	-0.59	0.44	-0.07	-0.57
85ILE	-0.02	-0.48	0.08	-0.03	-0.46
111GLN	-0.19	-0.20	0.03	0.00	-0.36
33SER	-0.40	-0.37	0.47	-0.07	-0.36
168ASP	-5.33	0.09	5.02	-0.14	-0.35
157ALA	0.00	-0.23	0.03	-0.01	-0.21
34GLY	-0.16	-0.23	0.23	-0.04	-0.19

Table S3. Top 15 energy-contributing residues in the 2αGG-MAPK13 system from residue energy decomposition analysis (kcal/mol).

Residue	ΔE_{COU}	ΔE_{vdw}	ΔE_{PB}	ΔE_{SA}	ΔG
110MET	-3.06	-1.19	1.80	-0.13	-2.58
108PRO	-3.40	-0.03	1.24	-0.02	-2.21
167LEU	-0.25	-1.37	0.15	-0.22	-1.70
107MET	-0.29	-1.00	0.16	-0.08	-1.22
39VAL	0.01	-1.09	0.20	-0.22	-1.10
31VAL	0.02	-1.02	0.22	-0.16	-0.94
85ILE	-0.12	-0.58	0.07	-0.04	-0.67
112THR	-0.46	-0.54	0.44	-0.03	-0.60
32GLY	-0.44	-0.46	0.44	-0.13	-0.59
173ARG	-0.55	-0.01	0.10	0.00	-0.45
160GLU	-0.51	-0.01	0.12	0.00	-0.40
111GLN	-0.31	-0.37	0.31	-0.02	-0.39
49GLU	-0.41	0.00	0.05	0.00	-0.37
109PHE	0.30	-0.98	0.42	-0.07	-0.33
116LYS	-0.56	-0.10	0.36	-0.01	-0.31

3. Hydrogen Bond Occupancies

Table S4. Top 10 Residues with Highest Hydrogen Bond Occupancy in HM-in Mode.

Donor	Acceptor	Occupancy%
flo@O6	PRO108@O	95.852
flo@O8	MET110@O	82.209
MET110@N	flo@O8	79.01
flo@O3	ASP168@OD1	54.873
flo@O3	ASP168@OD2	51.224
flo@O7	ASP113@OD2	45.477
flo@O7	ASP113@OD1	39.68
flo@O5	ASP113@OD2	26.937
flo@O4	SER33@O	23.938
flo@O5	ASP113@OD1	23.838

Table S5. Top 10 Residues with Highest Hydrogen Bond Occupancy in GRO-in Mode.

Donor	Acceptor	Occupancy%
flo@O4	PRO108@O	57.771
flo@O7	GLY32@O	12.994
MET110@N	flo@O3	11.294
flo@O7	ASP168@OD1	7.846
flo@O6	ASP113@OD1	7.496
flo@O3	PRO108@O	6.147
flo@O8	SER33@O	5.997
SER33@N	flo@O8	5.747
flo@O6	GLY32@O	5.297
flo@O6	SER33@O	4.648

Table S6. Top 10 Residues with Highest Hydrogen Bond Occupancy in 2αGG-MAPK13 system.

Donor	Acceptor	Occupancy%
gg@O4	PRO108@O	93.353
MET110@N	gg@O6	61.219
gg@O6	MET110@O	37.181
gg@O3	ASP168@OD1	22.889
gg@O5	SER33@O	15.692
gg@O3	ASP168@OD2	8.796
gg@O7	ASP113@OD2	6.347
SER33@N	gg@O7	5.997
gg@O7	ASP113@OD1	5.247
gg@O8	ASP113@OD1	3.798

4. Covariance Calculation

To clarify the origin of binding free energy fluctuation, the covariance between energy terms were calculated to quantify their interdependency. The total variance is given by:

$$\sigma^2{}_{\Delta G} = \sum_{i=1}^{n} \sigma^2{}_i + 2\sum_{i<j}^{n} Cov(E_i, E_j)$$

where E_i is the i-th energy contribution, σ_i is its standard deviation, and $Cov(E_i, E_j)$ is the covariance between E_i and E_j. The covariance is calculated as:

$$Cov(E_i, E_j) = \frac{1}{N-1}\sum_{k=1}^{N}(E_{i,k} - \bar{E}_i)\,(E_{j,k} - \bar{E}_j)$$

where N is the number of trajectory frames, $E_{i,k}$ is the instantaneous value of E_i at the k-th frame, and $\bar{E}_i$ is the mean value of E_i. The covariance analysis results are shown in Table S5.

Table S7. Covariance analysis results in HM-in mode (kcal^2/mol^2).

	ΔE_{COU}	ΔE_{vdw}	ΔE_{PB}	ΔE_{SA}
ΔE_{COU}	61.23	-12.64	-14.32	-0.04
ΔE_{vdw}	-12.64	7.63	0.66	0.02
ΔE_{PB}	-14.32	0.66	31.29	-0.16
ΔE_{SA}	-0.04	0.02	-0.16	0.01

Table S8. Covariance analysis results in 2αGG-MAPK13 system (kcal^2/mol^2).

	ΔE_{COU}	ΔE_{vdw}	ΔE_{PB}	ΔE_{SA}
ΔE_{COU}	65.09	-4.78	-25.72	-0.26
ΔE_{vdw}	-4.78	5.96	0.72	0.08
ΔE_{PB}	-25.72	0.72	30.14	-0.17
ΔE_{SA}	-0.26	0.08	-0.17	0.02

5. Computational Alanine Scanning (CAS)

Table S9. Top 15 energy-contributing residues in the HM-in conformation from alanine scanning and residue energy decomposition analysis (kcal/mol)

Residue	ΔE_{COU}	ΔE_{vdw}	ΔE_{PB}	ΔE_{SA}	ΔG
108PRO	-3.16	0.07	1.10	-0.02	-2.01
167LEU	0.30	-1.57	-0.08	-0.27	-1.62
109PHE	-0.93	-1.31	0.92	-0.06	-1.39
39VAL	-0.15	-1.23	0.20	-0.18	-1.36
110MET	-2.14	-1.30	2.84	-0.10	-0.70
107MET	0.13	-0.72	0.00	-0.07	-0.67
32GLY	-0.41	-0.52	0.43	-0.05	-0.55
157ALA	0.03	-0.55	0.03	-0.04	-0.53
31VAL	-0.03	-1.09	0.83	-0.20	-0.49
52ALA	-0.06	-0.44	0.18	-0.03	-0.35
72GLU	-0.05	-0.03	-0.27	0.00	-0.35
85ILE	0.11	-0.41	-0.02	-0.02	-0.34
34GLY	-0.07	-0.46	0.28	-0.05	-0.29
33SER	-0.16	-0.60	0.55	-0.08	-0.28
113D2A	0.43	-0.45	-0.13	-0.12	-0.26

6. SMD

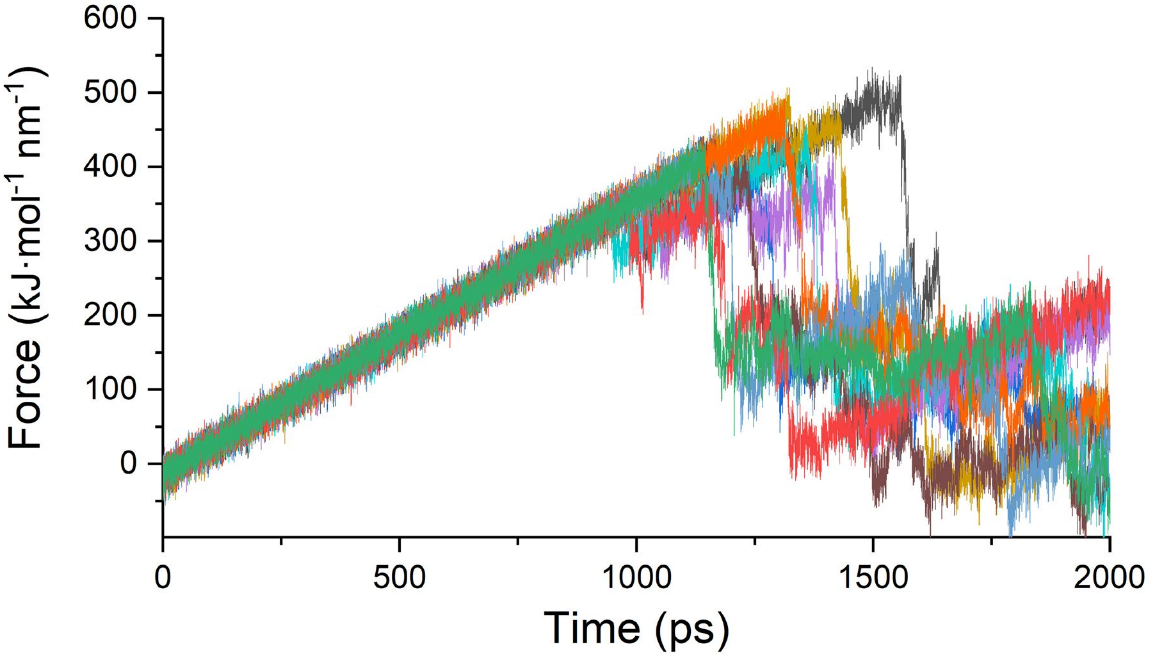

Figure S2. Force applied to floridoside within 10 SMD simulation.

7. Visualization data for binding mode in 2αGG-MAPK13 system

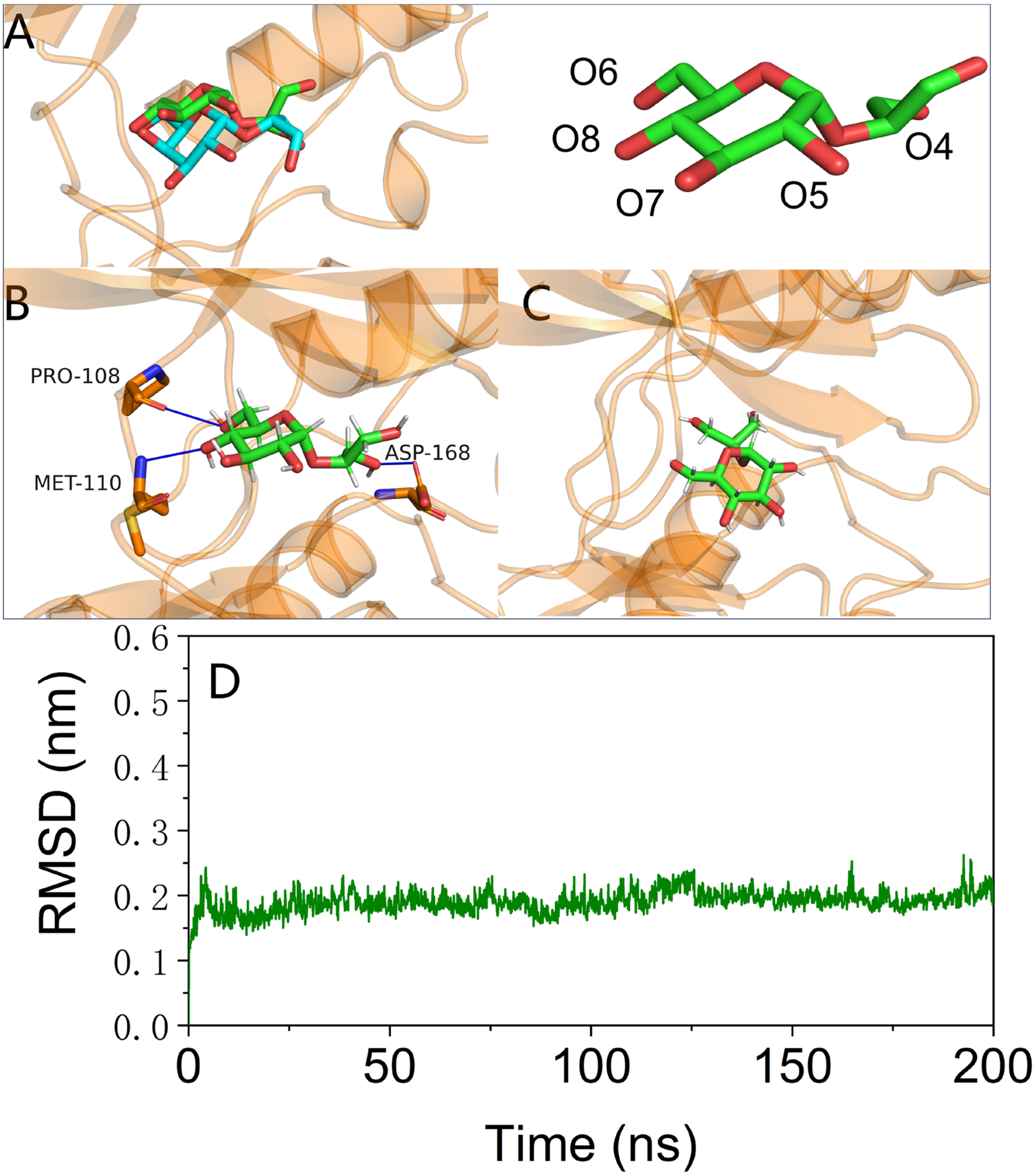

Figure S3. (A) Differences in docking results between 2αGG and floridoside. The skeleton of the floridoside is in blue, and 2αGG is in green. (B) Initial structure of the binding site and (C) average structure after conformational changes during simulation. (D) RMSD of the backbone in 2αGG-MAPK13 system.

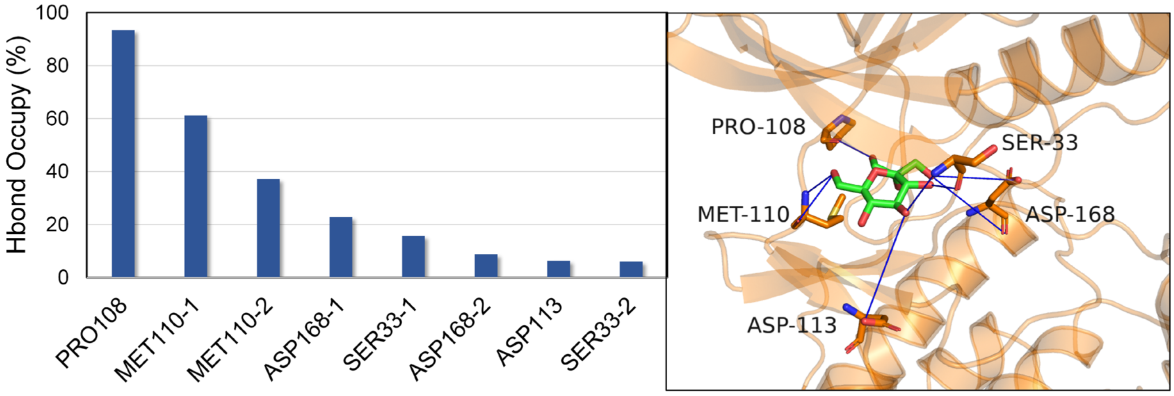

Figure S4. Hydrogen bond occupancy percentages in 2αGG-MAPK13 system. 3D schematic structures of binding region were extracted from the lowest-energy conformation, with blue dashed lines representing hydrogen bonds exhibiting higher temporal persistence. The dashed connectors solely indicate interatomic relationships and do not represent actual bonding patterns.

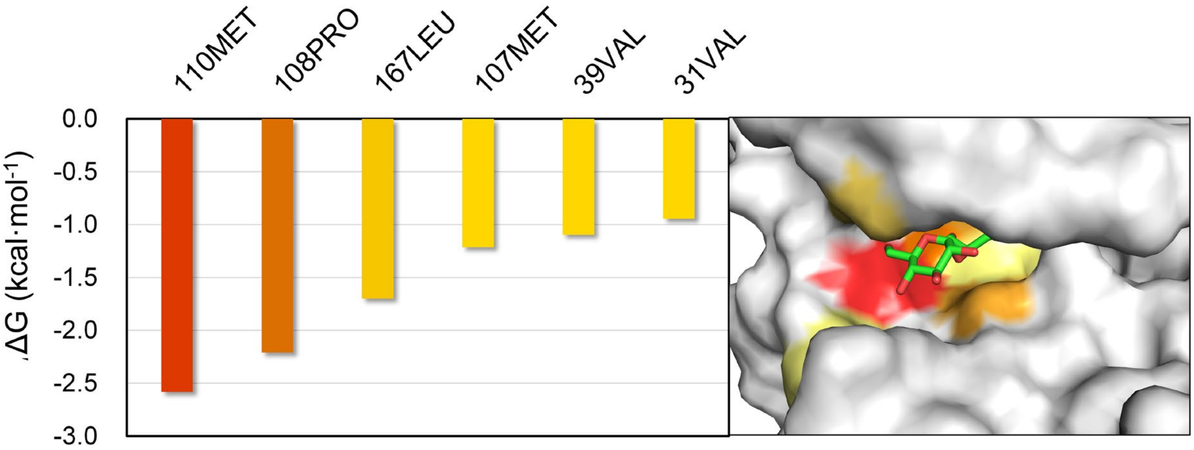

Figure S5. Residue energy decomposition of binding free energy in 2αGG-MAPK13 system. 3D schematic structures of binding region were extracted from the lowest-energy conformation and colored according to the corresponding residue energy intensities.

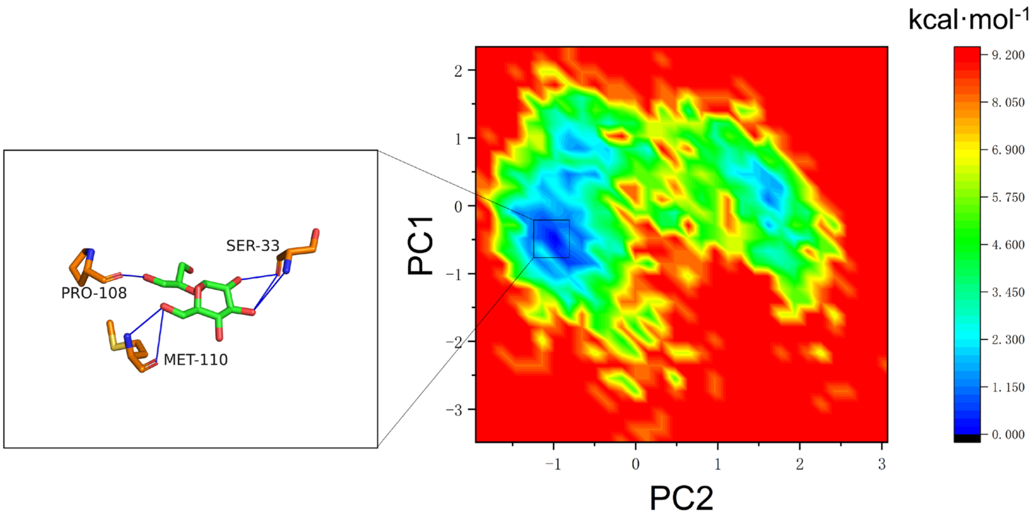

Figure S6. 2D free energy landscapes from MD trajectories for 2αGG-MAPK13 system, with blue-shaded regions indicating the lowest-energy conformations.

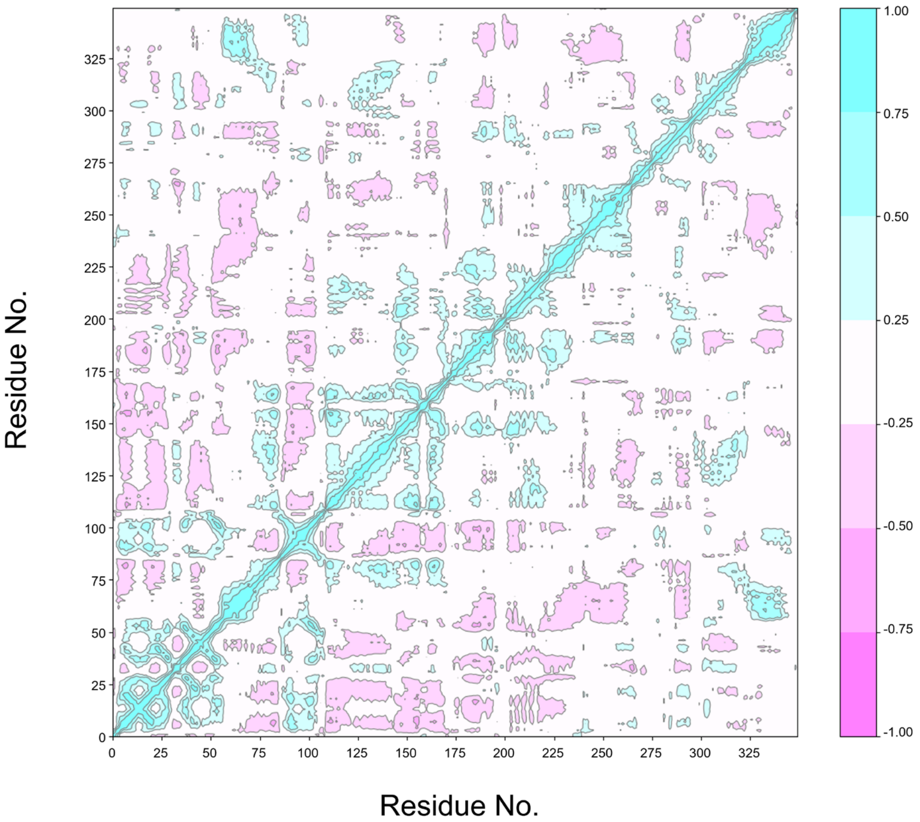

Figure S7. Cross-correlation matrixes for Cα atoms in 2αGG-MAPK13 system. Compared with the APO system, a redistribution of correlated motions was observed.

8. In Vitro Validation of MAPK13 Inhibition

Table S10. Inhibitory effect of Floridoside on MAPK13 activity measured by OD490. Data are presented as mean ± standard deviation (AVE ± SD, n = 3). Blank represents the background control without kinase; Maximum activity control represents the full kinase reaction without inhibitor.

		RLU			AVE	SD
Blank		57755	53531	50518	53934.67	2968.25
Maximum activity control		88272	86951	92566	89263.00	2397.03
Floridoside (nM)	100000.0000	55172	53713	52217	53700.67	1206.41
	25000.0000	56185	54664	55305	55171.00	717.01
	6250.0000	57153	55792	57638	56861.00	781.40
	1562.5000	58606	56880	59437	58307.67	1064.99
	390.6250	64883	58651	62429	61987.67	2563.27
	97.6563	67600	64256	62228	64694.67	2214.94
	24.4141	66714	70483	65039	67412.00	2276.65
	6.1035	83407	85681	77601	85210.00	1322.48
	1.5259	86542	93892	90004	90146.00	3002.30

9. Protein Structure Reliability Assessment

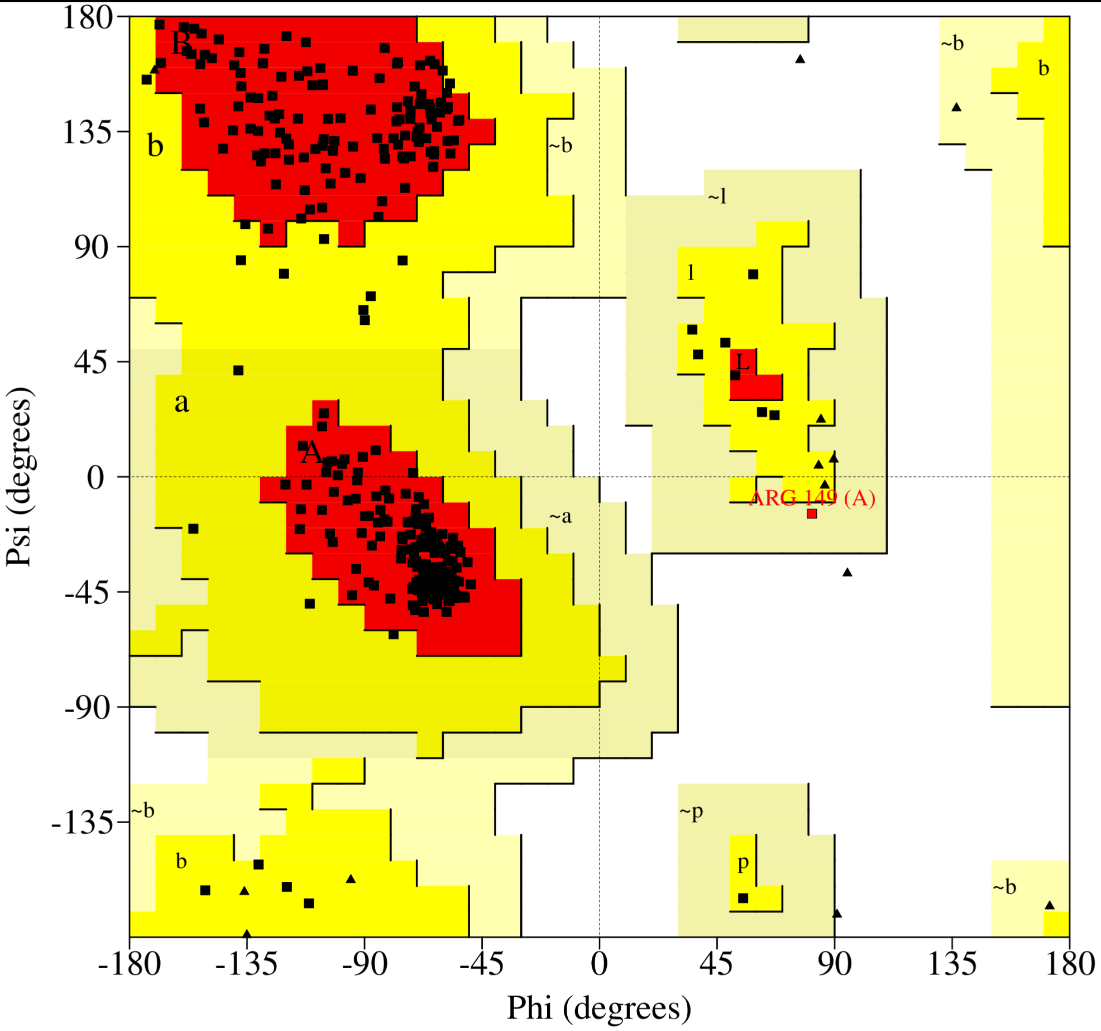

Plot statistics

Residues in most favoured regions [A,B,L]	289	91.7%
Residues in additional allowed regions [a,b,l,p]	25	7.9%
Residues in generously allowed regions [~a,~b,~l,~p]	1	0.3%
Residues in disallowed regions	0	0.0%
	----	------
Number of non-glycine and non-proline residues	315	100.0%
Number of end-residues (excl. Gly and Pro)	1	
Number of glycine residues (shown as triangles)	17	
Number of proline residues	17	

Total number of residues	350	

Figure S8. Ramachandran plot of the MAPK13 structure with repaired residues. 91.7% of the residues in the repaired model are located within the most favored regions, with 0% in the disallowed regions.

10. Structure

HM-in conformation

35

O	-10.0330	10.8270	-32.6980
C	-7.5900	12.3280	-32.4450
C	-8.8870	12.9740	-32.9380
C	-7.6010	10.8280	-32.7420
C	-10.1020	12.2250	-32.3740
C	-8.8680	10.1760	-32.1840
O	-8.8390	10.1870	-30.7570
C	-9.2160	8.9270	-30.2240
C	-10.2890	9.1620	-29.1700
O	-9.6630	9.3390	-27.9040
H	-9.2450	10.2180	-27.9040
C	-7.9810	8.2630	-29.6230
O	-7.0010	9.2490	-29.3200
H	-6.1610	8.7860	-29.1620
O	-6.4570	10.2020	-32.1670
H	-6.7560	9.3840	-31.7350
C	-11.4200	12.7530	-32.9300
O	-11.2770	14.1380	-33.1920
H	-12.1690	14.5090	-33.3060
O	-6.4700	12.9270	-33.0910
H	-5.8890	12.2080	-33.3920
O	-8.9340	12.9210	-34.3660
H	-8.9130	13.8310	-34.7080
H	-7.5152	12.4789	-31.3883
H	-8.9115	13.9909	-32.6061
H	-7.5811	10.7004	-33.8042
H	-10.0747	12.3784	-31.3154
H	-8.9067	9.1547	-32.5008
H	-9.6102	8.2810	-30.9804
H	-10.9449	8.3175	-29.1311
H	-10.8555	10.0350	-29.4187
H	-7.5764	7.5645	-30.3253
H	-8.2573	7.7526	-28.7241
H	-12.2004	12.6002	-32.2141
H	-11.6707	12.2342	-33.8316

HM-out conformation

35

O	-8.9490	11.1360	-32.3270
C	-11.4300	12.4280	-33.0060
C	-10.3380	12.1760	-34.0490
C	-10.8140	12.5240	-31.6100
C	-9.4880	10.9640	-33.6470
C	-9.9480	11.2970	-31.3160
O	-10.7670	10.1350	-31.1900
C	-10.3760	9.3460	-30.0780
C	-10.7920	7.9080	-30.3510
O	-11.6550	7.4700	-29.3050
H	-11.4700	8.0190	-28.5240
C	-11.0540	9.8950	-28.8270
O	-10.2830	9.5650	-27.6760
H	-9.6210	8.9070	-27.9480
O	-11.8370	12.6250	-30.6240
H	-11.4740	13.1260	-29.8740
C	-8.3110	10.7420	-34.5900
O	-7.2090	11.5010	-34.1250
H	-6.6040	10.8920	-33.6680
O	-12.1160	13.6420	-33.3010
H	-12.0660	13.7780	-34.2630
O	-9.4910	13.3250	-34.1410
H	-9.2680	13.4680	-35.0770
H	-12.1210	11.6120	-33.0330
H	-10.8030	11.9850	-34.9930
H	-10.2010	13.4000	-31.5780
H	-10.1440	10.1200	-33.6890
H	-9.4410	11.4410	-30.3850
H	-9.3170	9.3770	-29.9260
H	-9.9240	7.2830	-30.3850
H	-11.3020	7.8510	-31.2900
H	-12.0300	9.4670	-28.7350
H	-11.1330	10.9590	-28.9060
H	-8.5760	11.0590	-35.5770
H	-8.0540	9.7030	-34.6160

GRO-in conformation

35

O	-8.0310	9.9000	-31.3170
C	-5.6390	10.2550	-32.8780
C	-5.9050	8.9720	-32.0880
C	-6.3810	11.4310	-32.2400

C	-7.4140	8.7530	-31.9210
C	-7.8660	11.1040	-32.0720
O	-8.5000	11.0220	-33.3480
C	-9.7180	11.7480	-33.3670
C	-9.7680	12.5510	-34.6590
O	-10.3330	13.8300	-34.3880
H	-10.2210	14.0040	-33.4370
C	-9.7630	12.6580	-32.1440
O	-11.1060	12.7930	-31.6910
H	-11.5740	13.3540	-32.3340
O	-6.2440	12.5970	-33.0460
H	-5.9270	13.3150	-32.4730
C	-7.7330	7.5510	-31.0390
O	-7.8210	7.9890	-29.6940
H	-6.9380	8.2950	-29.4260
O	-4.2420	10.5360	-32.8970
H	-4.0260	10.9980	-32.0680
O	-5.3080	9.0750	-30.7930
H	-4.4320	8.6540	-30.8240
H	-5.9889	10.1164	-33.8796
H	-5.4835	8.1470	-32.6234
H	-5.9514	11.6115	-31.2768
H	-7.7955	8.5833	-32.9062
H	-8.3389	11.8922	-31.5243
H	-10.5658	11.0962	-33.3313
H	-10.3715	12.0371	-35.3777
H	-8.7791	12.6681	-35.0506
H	-9.3775	13.6217	-32.4042
H	-9.1689	12.2300	-31.3638
H	-6.9565	6.8205	-31.1303
H	-8.6599	7.1106	-31.3419

2αGG

35

O	-10.8950	10.4070	-32.7910
C	-8.4190	10.9960	-34.0330
C	-9.6750	11.8200	-34.3100
C	-8.7670	9.5740	-33.5790
C	-10.5200	11.7770	-33.0440
C	-9.8150	9.5780	-32.4550
O	-9.1750	9.9970	-31.2720
C	-9.4680	9.2020	-30.1070
C	-9.8650	7.7900	-30.5470
O	-10.4190	6.9900	-29.5390
H	-10.6790	6.1150	-29.8820
C	-10.5720	9.9050	-29.3180
O	-10.5540	9.5510	-27.9550
H	-10.5180	10.3790	-27.4550
O	-7.5990	8.8600	-33.2250
H	-7.0780	8.7770	-34.0380
C	-11.8100	12.5710	-33.0460
O	-11.5160	13.9650	-33.0500
H	-12.1960	14.3760	-33.6040
O	-7.6350	10.9750	-35.2080
H	-8.2420	11.0620	-35.9570
O	-9.3620	13.1580	-34.6100
H	-8.9980	13.5500	-33.8030
H	-7.8678	11.4456	-33.2336
H	-10.1924	11.4148	-35.1544
H	-9.2142	9.0615	-34.4050
H	-9.8921	12.2250	-32.3024
H	-10.2198	8.5982	-32.3098
H	-8.6136	9.1034	-29.4705
H	-8.9892	7.2990	-30.9169
H	-10.6286	7.9107	-31.2868
H	-11.5200	9.6329	-29.7330
H	-10.4073	10.9600	-29.3875
H	-12.3775	12.3297	-32.1716
H	-12.3756	12.3257	-33.9205
